# Disordered testosterone transport in mice lacking the ganglioside GM2/GD2 synthase gene

**DOI:** 10.1002/2211-5463.13603

**Published:** 2023-04-06

**Authors:** Koichi Furukawa, Kogo Takamiya, Yuhsuke Ohmi, Robiul H. Bhuiyan, Orie Tajima, Keiko Furukawa

**Affiliations:** ^1^ Department of Biomedical Sciences Chubu University College of Life and Health Sciences Kasugai Japan; ^2^ Department of Molecular Biology and Biochemistry Nagoya University Graduate School of Medicine Japan; ^3^ Department of Neuroscience University of Miyazaki Faculty of Medicine Japan; ^4^ Department of Clinical Engineering Chubu University College of Life and Health Sciences Kasugai Japan; ^5^ Department of Biochemistry and Molecular Biology University of Chittagong Chittagong Bangladesh

**Keywords:** aspermatogenesis, ganglioside, knockout, male infertility, testis, testosterone

## Abstract

Genetic disruption of glycosyltransferases has provided clear information on the roles of their reaction products in the body. Our group has studied the function of glycosphingolipids by genetic engineering of glycosyltransferases in cell culture and in mice, which has demonstrated both expected and unexpected results. Among these findings, aspermatogenesis in ganglioside GM2/GD2 synthase knockout mice was one of the most surprising and intriguing results. There were no sperms in testis, and multinuclear giant cells were detected instead of spermatids. Although serum levels of testosterone in the male mice were extremely low, testosterone accumulated in the interstitial tissues, including Leydig cells, and seemed not to be transferred into the seminiferous tubules or vascular cavity from Leydig cells. This was considered to be the cause of aspermatogenesis and low serum levels of testosterone. Patients with a mutant GM2/GD2 synthase gene (SPG26) showed similar clinical signs, not only in terms of the neurological aspects, but also in the male reproductive system. The mechanisms for testosterone transport by gangliosides are discussed here based on our own results and reports from other laboratories.

## Graphical abstract

GM2/GD2 synthase knockout mice showed aspermatogenesis. No sperm were detected in testis, but multinuclear giant cells were observed. Although serum testosterone levels were low, testosterone accumulated in interstitial Leydig cells, and seemed not to be transferred into seminiferous tubules or the vascular cavity, leading to low serum levels and aspermatogenesis. Patients with mutant GM2/GD2 synthase gene showed similar clinical signs.

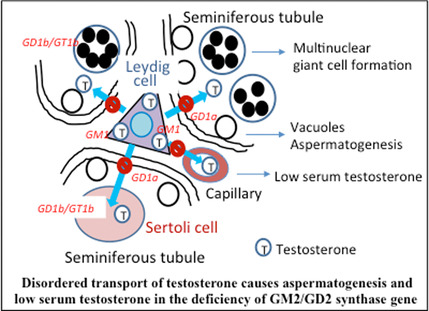

**Abbreviations**
FSH: follicle‐stimulating hormone
GM2/GD2 synthase: β1,4‐*N*‐acetylgalactosaminyltransferase (B4galnt1)
GM3 synthase: α2,3‐sialyltransferase (St3gal5)
KO: knockout

Gangliosides (sialic acid‐containing glycosphingolipids) are expressed in various tissues and cells of vertebrates [1, 2] and are considered to be involved in the regulation of development, differentiation, and activation of individual tissues/cells [2, 3]. In particular, gangliosides are considered to regulate neuronal differentiation and function in vertebrates, since they are highly expressed in the nervous tissues of vertebrates with similar composition among different species [4]. Several studies have examined the biochemical and pathological aspects of gangliosides in normal tissues [5] and various diseases [6]. Aberrant expression of some gangliosides has been also reported in malignantly transformed cells and under pathological conditions [6, 7]. The successful molecular cloning of glycosyltransferase genes responsible for the synthesis of glycosphingolipids [8] has facilitated the elucidation of the roles of gangliosides in the regulation/maintenance of functions of various tissues and cells [9]. In particular, the significant roles of different glycosphingolipids have been investigated and elucidated using gene knockout (KO) mice, leading to the discovery of unexpected findings [10].

Among the new findings discovered using KO mice, disordered secretion and transport of hormones are particularly intriguing [11, 12]. Substantial attention has been paid to the role of gangliosides in the nervous system and in neurological abnormal phenotypes [12]. We observed neurodegeneration in double KO mice of *B4galnt1* and *St8sia1*, which was mainly due to complement activation [13]. At this stage, we demonstrated various degree of neuronal disorders in the KO mouse series depending on the degree of the defects in the synthetic profiles [14]. However, there are few reports on the disordered secretion and transport of various hormones [10].

Following progress in the generation and analysis of gene KO mice lacking certain glycosphingolipids, clinical cases due to deficient ganglioside synthase genes were reported, for example, GM3 synthase deficiency [15, 16, 17, 18] and GM2/GD2 synthase deficiency [19, 20, 21, 22, 23]. Clinical information about these patients reflects the various “unexpected phenotypes” that the individual KO mice exhibited. In this review, we have summarized important findings concerning disordered secretion and transport of hormones based on the deficiency of gangliosides, based mainly on our own experimental results but also including data from other research groups.

## The ganglioside synthetic pathway and the use of gene knockouts to investigate ganglioside functions

**Fig. 1:**
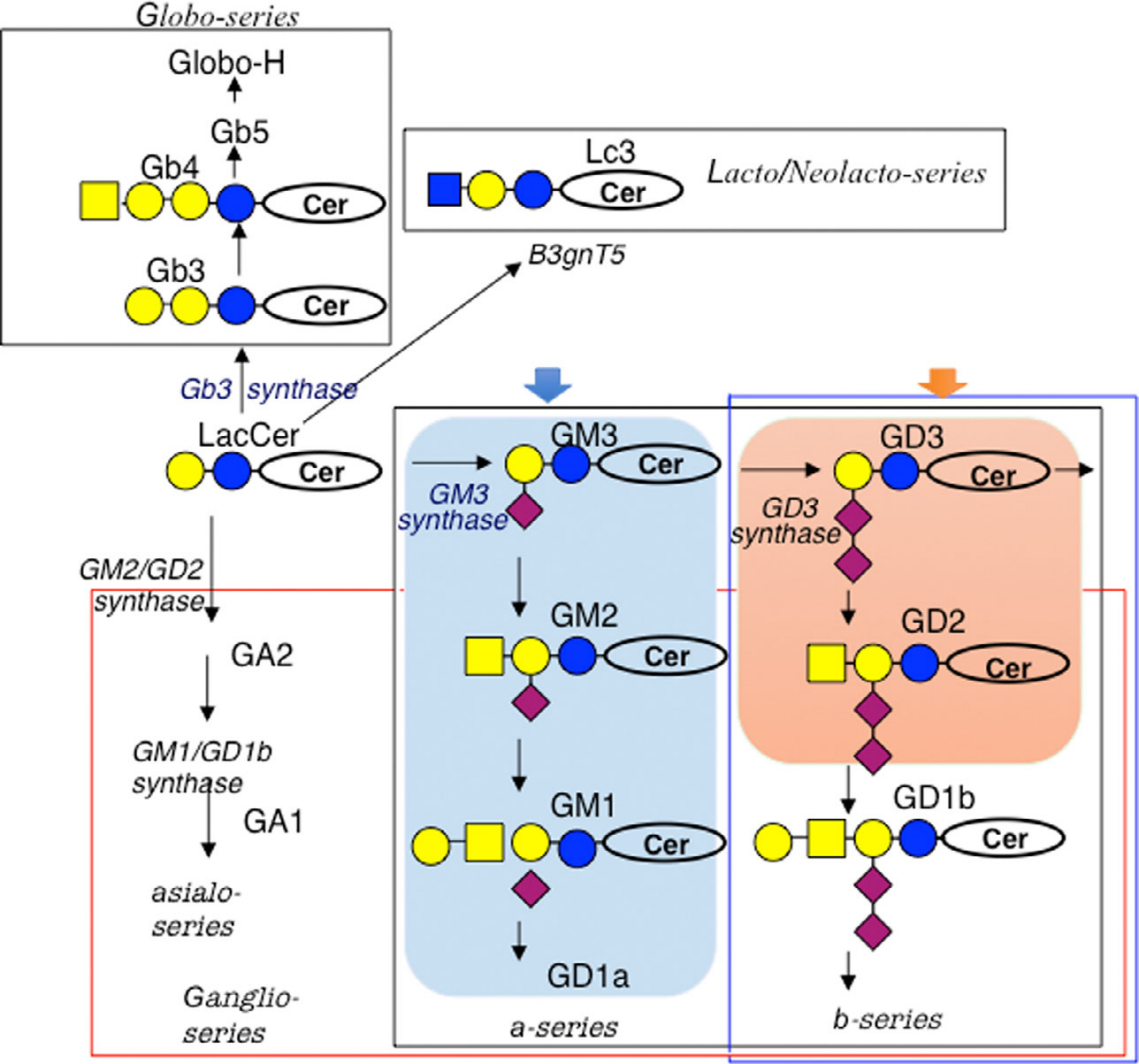
Synthetic pathways of gangliosides. The majority of glycosphingolipids are synthesized from LacCer. GM2/GD2 synthase is a key enzyme for the synthesis of all complex gangliosides. Deleted structures in KO mice are indicated by squares.

Figure 1 shows the synthetic pathway of glycosphingolipids starting from glucosylceramide/galactosyl ceramide. The majority of glycolipids are synthesized via glucosylceramide followed by β1,4‐galactose substitution, forming lactosylceramide (LacCer). LacCer is used in several diverse pathways, for example, the ganglioside series, the globo‐series, and the lacto/neolacto‐series. Gangliosides can be further divided into a‐series, b‐series, c‐series, and asialo‐ (0‐) series. As shown in Fig. 1, GM3 synthase, GD3 synthase, and GM2/GD2 synthase are involved in the synthesis of core gangliosides, through which more complex species are formed. Therefore, disruption of some of these glycosyltransferase genes results in the complete loss of all structures of glycolipids located in the individual synthetic pathways. The outline of the features of various KO mice was reviewed in our recent article [10]. Briefly, the KO mouse lines exhibited increasingly serious phenotypes depending on the degree of ganglioside defects [14].

## Male sterility in complex ganglioside‐lacking mice

GM2/GD2 synthase gene KO mice exhibited no marked abnormal phenotypes during the early stage of life after birth [24]. This result was different from what many researchers expected at the time. However, these KO mice showed gradually progressing neurodegeneration with aging [25]. The only abnormal finding we detected in the initial screening of the KO mice was a delay in the neurotransmission [24]. However, during long‐term observation, they exhibited abnormal sensory, and then motor nerve dysfunctions, as well as pathological disorders [25]. One of the most serious issues was male infertility in the KO mice lacking complex gangliosides [26].

**Fig. 2:**
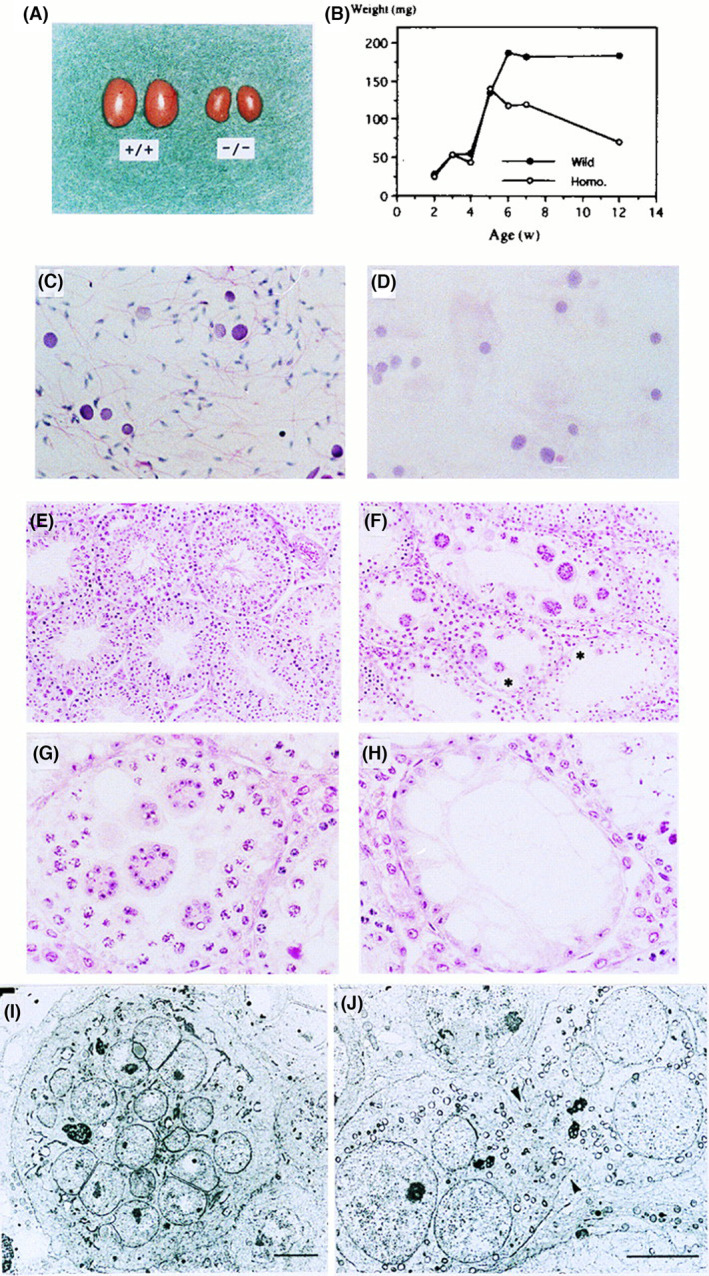
Aspermatogenesis and male sterility in GM2/GD2 synthase KO mice. Morphology and growth of the testis of WT mice and the KO mice. (A) Eight‐week‐old WT (+/+) and the KO (−/−) testes. A bar indicates 5 mm. (B) Changes in testicular weight in WT and KO mice. (C, D) Smear of seminiferous fluid from WT (C) and KO (D) mice. (Hematoxylin/eosin) (E, F) Histopathology of testis from 10‐week‐old WT (E) and KO (F) mice. (H/e) (G, H) High magnification of KO mice testis (H/e) Note the diffuse vacuoles in Sertoli cells. Bars in C, D, G, and H indicate 25 μm, and bars in E and F indicate 50 μm. (I, J) Electron micrographs of multinuclear giant cells. A giant cell (I) and an unseparated prematurely opened intercellular bridge (arrows) (J) are shown. Bars indicate 5 μm. This figure is reproduced from our previous paper [26].

The main features of the male infertility in B4galnt1 KO mice were as follows: (a) disappearance of sperms in the smear of testis (Fig. 2A–D); (b) extremely low serum levels of testosterone (KO: 21.0 ± 4.10 ng·dL^−1^ vs WT: 386.63 ± 111.90 ng·dL^−1^); (c) no spermatozoa and polynuclear giant cells in the seminiferous tubules (Fig. 2E–J); (d) accumulation of testosterone in the interstitial Leydig cells (Fig. 4); and (e) lowered excretion of intratesticularly injected testosterone and its restoration by co‐injection with complex gangliosides (Fig. 5). All these findings suggested that transportation of testosterone from Leydig cells into seminiferous tubules via basement membrane is disturbed by the lack of gangliosides in various cells in testicular tissues. The passage of testosterone via vascular membrane should be also disturbed by ganglioside deficiency, resulting in extremely low levels in the serum.

## Ganglioside expression in testicular tissues and involvement in testosterone transport

**Fig. 3:**
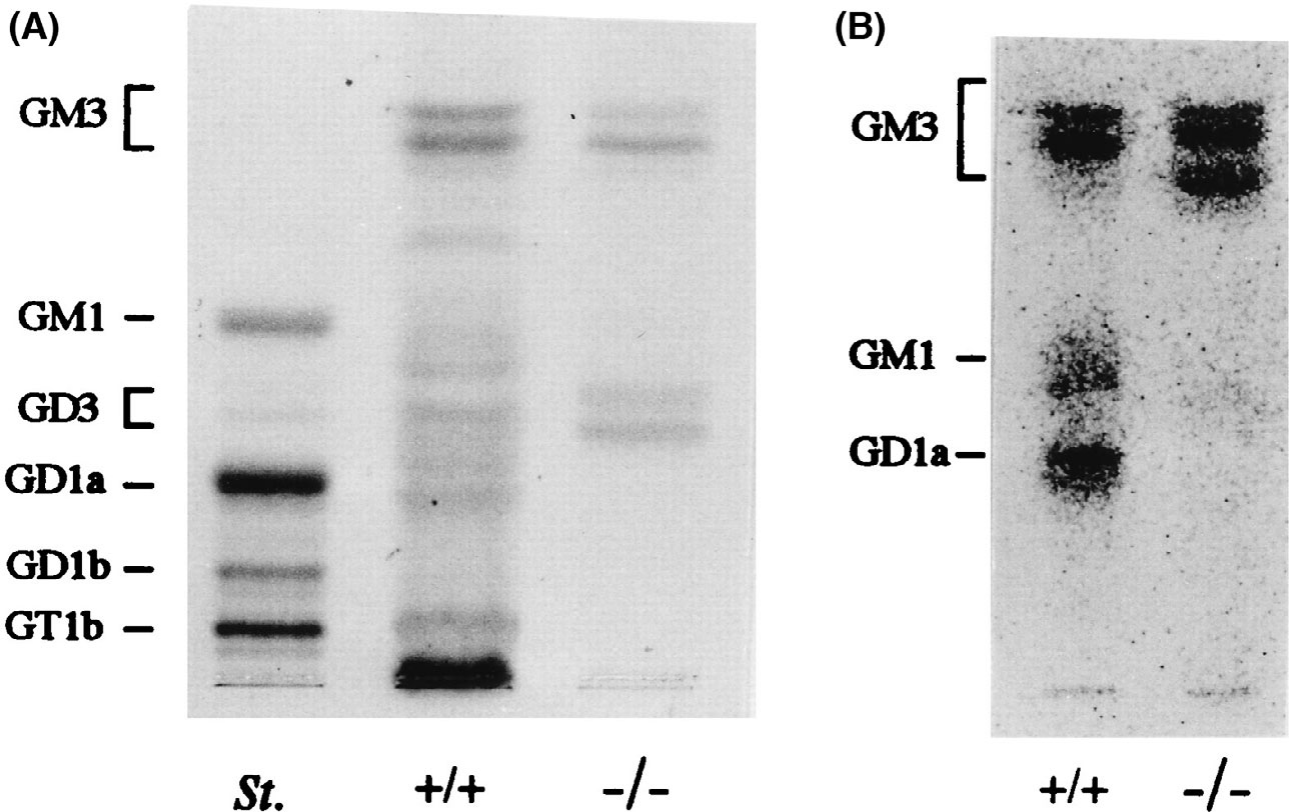
Expression profiles of gangliosides in murine testis. Expression of gangliosides in testis. (A) TLC of acidic glycosphingolipids from testis. St., bovine brain ganglioside mixture as a standard. Acidic glycolipids were separated by TLC with a solvent (chloroform : methanol : 0.2% CaCl_2_, 55 : 45 : 10). Resorcinol spray was used for detection. (B) Profiles of acidic glycosphingolipids in nongerm cells. Nongerm cells from testis were cultured primarily after elimination of germ cells, then metabolically labeled with [^14^C]glucosamine and analyzed for acidic glycolipids by TLC as in A. This figure is reproduced from our previous paper [26].

As shown in Fig. 3A, the extracts from the testis of WT mice showed multiple ganglioside components containing GM3, GM1, GD3, GD1a, and GT1b. Generally, this profile corresponds with ovine testis [27]. On the contrary, those of the KO mice showed two bands of GM3 and GD3. In TLC of [^14^C]glucosamine‐labeled nongerm cell culture, three components, GM3, GM1, and GD1a, were detected in the WT sample, while strong GM3 and a faint GD3 bands were detected in the KO sample (Fig. 3B). Thus, b‐series gangliosides such as GD3, GD1b, and GT1b were considered to be derived from germ cells. These ganglioside profiles of testis seem to be similar with those of brain tissues [24]. Immunohistostaining of the testis tissues revealed that GT1b was expressed only in the intermediate layer of germ cells, GD1b was in all germ cells, and GD1a was at the edge of the seminiferous tubules, possibly in Sertoli cells (data not shown). Consequently, gangliosides are differentially distributed in WT testis, for example, GT1b and GD1b are mainly in germ cells, GD1a is in Sertoli cells, and GM1 is in Leydig cells.

## Distribution and transport of testosterone in mouse testis

**Fig. 4:**
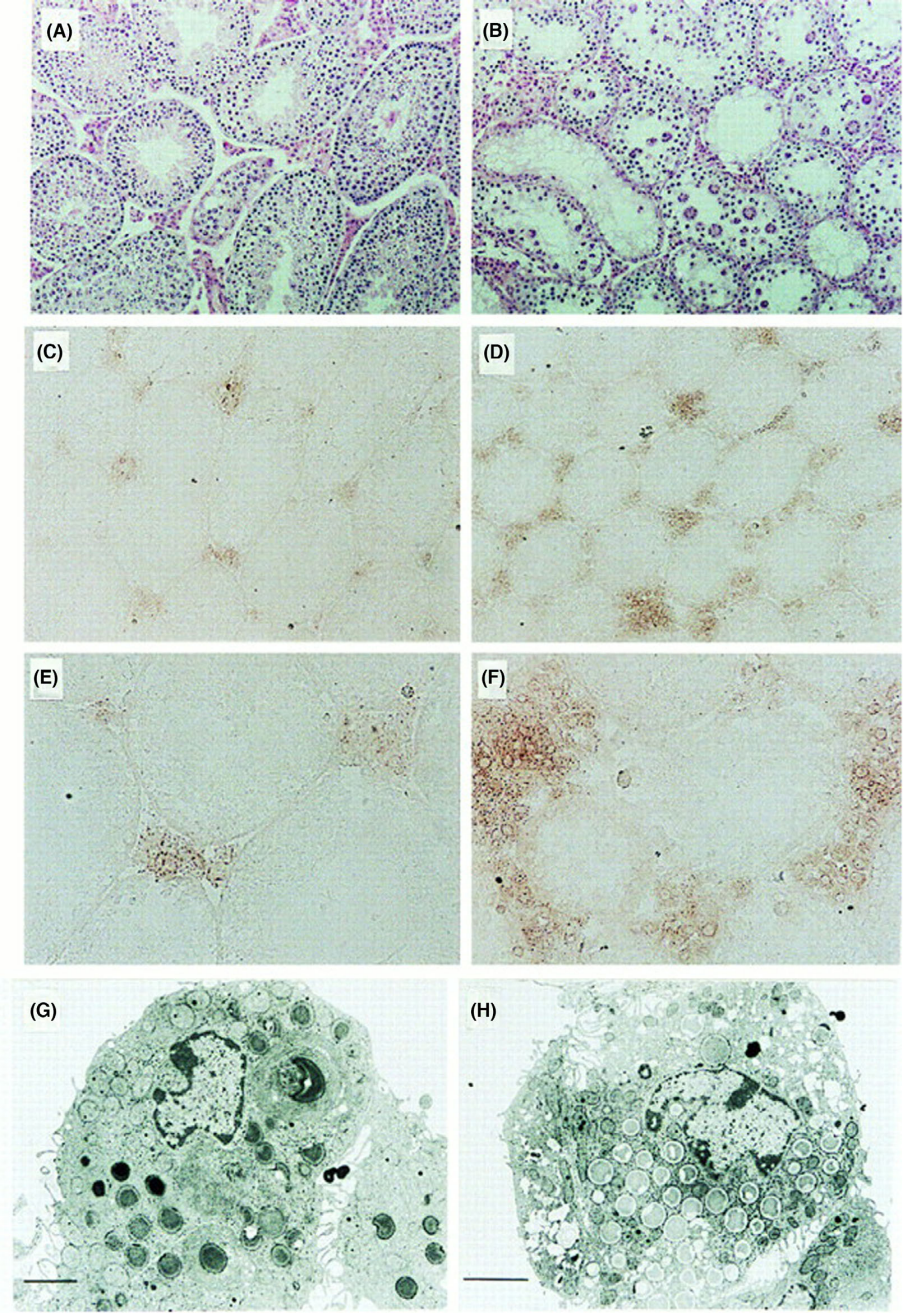
Accumulation of testosterone in the interstitial cells of GM2/GD2 synthase KO mice. Testosterone production in the interstitial cells of testis. (A, B) Hematoxylin/eosin staining of testis from wild‐type (A) and the KO (B) mice. (C–F) Immunohistochemistry for testosterone with polyclonal antibody in wild‐type (C, E) and the KO (D, F) testis. Bars in A–D indicate 50 μm, Bars in E and F indicate 25 μm. (G, H) Electron micrograph of Leydig cells of wild‐type (G) and the KO (H) mice. Bars indicate 2 μm. This figure is reproduced from our previous paper [26].

Since serum levels of testosterone were extremely low in the KO mice, we performed immunostaining of testis using anti‐testosterone antibody. As compared with HE staining patterns in Fig. 4A,B, testosterone accumulated to a greater extent in interstitial Leydig cells of the KO mice than those of WT mice (Fig. 4C–F). These results suggested that testosterone transport from Leydig cells into serum and seminiferous tubules is blocked. Leydig cells containing a number of granules with low density were frequently detected in the KO testis (Fig. 4G,H).

**Fig. 5:**
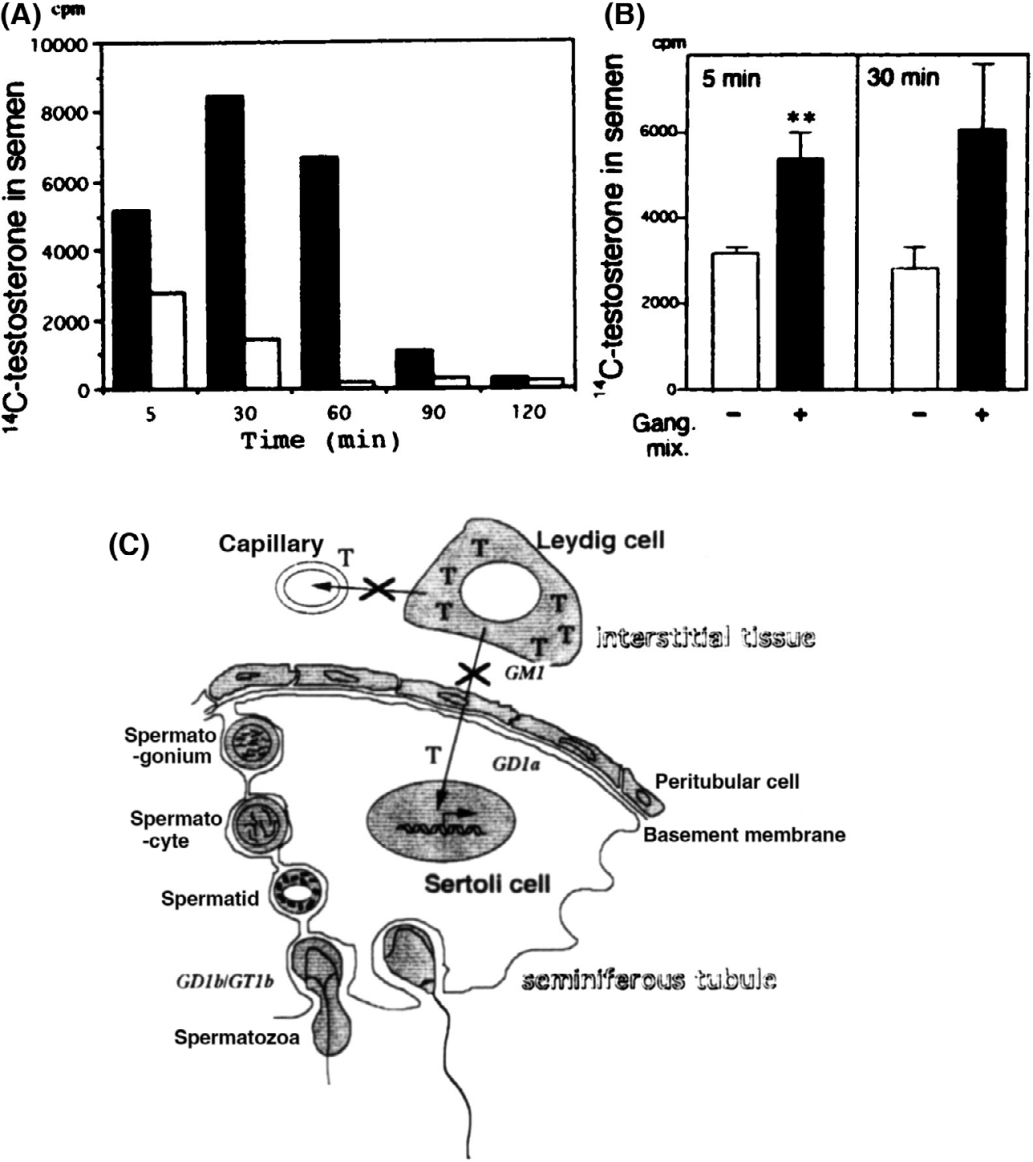
Disrupted excretion of testosterone from seminiferous tubules and restoration by addition of gangliosides. (A) Transport of intratesticularly injected [^14^C]testosterone into semen. The radioactivity in semen after injection was measured at the time points indicated. These experiments were repeated at least three times and showed essentially the same results. A representative result is given. Black bars, WT; white bars, KO. (B) Improvement of [^14^C]testosterone excretion in the presence of gangliosides in the KO mice. [^14^C]testosterone was injected as in A, with or without gangliosides (10 μg), then cpm in semen was examined at 5 and 30 min after injection. *n* = 3 for each group. Means ± SD are presented. Results were analyzed with a Student's two‐tailed *t*‐test. **, *P* < 0.05. (C) A scheme to show the essential role of complex gangliosides in testosterone transport from Leydig cells in testis. T, testosterone. An X superimposed on an arrow represents disrupted transport. This figure is reproduced from our previous paper [26].

To examine testosterone transport in the testis, excreted [^14^C]testosterone in semen was measured after intratesticular injection. As shown in Fig. 5A,B, the permeability of [^14^C]testosterone from interstitial tissue to seminiferous tubules was strongly reduced in the KO mice compared with the secretion pattern of WT mice (Fig. 5A). When gangliosides were co‐injected with testosterone in the KO mice, secretion of [^14^C]testosterone was markedly improved (Fig. 5B).

Although complex gangliosides have been thought to be co‐receptors of peptide hormones such as follicle‐stimulating hormone (FSH) [3, 5], cultured Sertoli cells from the KO mice responded as well to FSH as those from WT mice. Therefore, disrupted hormonal signaling due to ganglioside deficiency does not seem to be a mechanism for the disorders in the KO mouse testis.

The multinuclear giant cells in the KO mice might be also associated with other factors [28] such as cytochalasin D treatment [29]. Therefore, it seemed difficult to specify the cause of aspermatogenesis based on morphological findings. According to Matikainen *et al*. [30], the completion of meiosis and spermiogenesis supported by FSH depended on androgens from Leydig cells in hypophysectomized rats. If so, it seems likely that the testosterone produced in the Leydig cells could not reach the Sertoli cells in seminiferous tubules or the blood stream because of the disruption of the testosterone transport pathway, leading to the degeneration of immature round spermatids and spermatocytes and to the formation of multinuclear giant cells (Fig. 5C). The results shown in Fig. 5A,B for the suppressed secretion of [^14^C]testosterone, and also increased secretion after co‐injection with complex gangliosides, support this interpretation. The poor transport of testosterone in the KO mice might also affect development of other tissues such as muscles and some parts of brain. This was previously reported as a sexually dimorphic nucleus in the preoptic area in rats (data not shown) [31].

Insufficient growth of testis was found from 4 to 5 weeks of age (Fig. 2A,B), when gonads and sex maturation progress under hormonal control in the WT mice. Although it is suggested that disturbed testosterone flow is a major cause of aspermatogenesis in the KO mice, the effects of lack of complex gangliosides might affect pathways other than testosterone flow, since the testicular feminized mutant mice lacking androgen receptor show similar abnormal features with attenuated severity of symptoms [32].

Despite disturbed testosterone transport to seminiferous tubules, spermatogonia/spermatocytes, but no spermatids, seem to be present in the KO mouse testis, as indicated by the expression of a “spermatogenesis” marker, KIF11 [33].

## The mechanism of action of gangliosides in testosterone transport

Glycosphingolipids are expressed mainly on the surface of the cell membrane. Since they are embedded in the outer layer of the cell membrane, they have no cytoplasmic domains that transduce cell signals into cells [34]. Therefore, we need to suppose that gangliosides play roles by forming molecular complexes with cell surface functional molecules such as growth factor receptors and/or integrins [35]. In order to identify interacting membrane molecules, we performed enzyme‐mediated activation of radical sources/mass spectrometry [36], and reported interesting results [37]. There are a few studies on the roles of gangliosides in mouse embryogenesis and embryonic stem cell differentiation [38]. However, these results of these studies struggle to explain the roles of gangliosides in testosterone transport. We examined the binding of testosterone with various gangliosides expressed on individual cells in testis, and showed specific binding with gradual intensity depending on the numbers of sialic acids [26]. Thus, testosterone might be transferred to the cell membrane from cell to cell. The actual mechanisms for this transport of testosterone remain to be clarified.

## Clinical abnormalities in the reproductive system of SPG26 patients

There have been five studies of patients with *B4GALNT1* gene mutations, and the majority of the patients (33 cases, 14 families, and 15 mutations) showed clinical features of neurodegeneration [19, 20, 21, 22, 23]. The disease onset is in childhood with developmental delay, and progressive spasticity of the legs is a universal feature leading to gait impairment. Speech is characterized by mild‐to‐moderate dysarthria, and there is intellectual impairment in most patients in the mild‐to‐moderate range [22]. When the genetic mutation is found in *B4GALNT1*, this can be diagnosed as *B4GALNT1‐*associated hereditary spastic paraplegia (SPG26) [23], while the responsible gene in the first case was found to be present on chromosome 12 in 2005 [39]. The enzyme activities of the mutated *B4GALNT1* gene and patients' profiles were previously reported by our group [40]. The onset of abnormal signs and symptoms is generally later than those observed in patients with *ST3GALT5* gene deficiency [15, 16, 17, 18]. These findings in the *B4GALNT1* mutation cases correspond well with abnormal phenotypes observed in the *B4galnt1* KO mice [24, 26]. Among all male patients, infertility/low testosterone were observed in three of 17 male SPG26 patients [19, 20, 21, 22, 23]. Taking the cases that have no data available into consideration, it appears that *B4GALNT1* deficiency may generally induce male infertility in humans as well. As shown in the KO mice, no disordered reproductive system could be found in the human female cases.

## Perspectives

At present, there is no efficient therapeutic method of treating aspermatogenesis or general systemic disorders of SPG26 patients. Local administration of gangliosides toward diseased sites may possibly be an easy and safe approach [41]. The use of exogenous gangliosides to reduce ROS‐induced changes in human spermatozoa has been studied [42, 43]. The effects of gangliosides have also been examined not only for spermatozoa, but also for oocytes and preimplantation embryos [44]. Therapeutic injection of a *B4GALNT1*‐expression vector of adeno‐associated virus might be a promising way to treat the disease, but how and when we should administer these reagents needs to be carefully investigated.

## Conflict of interest

The authors declare no conflict of interest.

## Author contributions

KoF and KT wrote the manuscript and made a general plan for this project. KT, YO, OT, RHB, and KeF generated original figures based on their experimental contributions.

## Data Availability

All data are available. Koichi Furukawa should be contacted if someone wants to request the data from this study.
